# Electrotonic Coupling between Pyramidal Neurons in the Neocortex

**DOI:** 10.1371/journal.pone.0010253

**Published:** 2010-04-26

**Authors:** Yun Wang, Amey Barakat, Hongwei Zhou

**Affiliations:** 1 Caritas St. Elizabeth's Medical Center, Tufts University, Boston, MA 02135, USA; 2 School of Optometry & Ophthalmology, Wenzhou Medical College, Wenzhou, Zhejiang, 325027, P.R.China; INSERM U862, France

## Abstract

Electrotonic couplings (i.e., electrical synapses or gap junctions) are fundamental to neuronal synchronization, and thus essential for many physiological functions and pathological disorders. Interneuron electrical synapses have been studied intensively. Although studies on electrotonic couplings between pyramidal cells (PCs) are emerging, particularly in the hippocampus, evidence is still rare in the neocortex. The electrotonic coupling of PCs in the neocortex is therefore largely unknown in terms of electrophysiological, anatomical and synaptological properties. Using multiple patch-clamp recording with differential interference contrast infrared videomicroscopy (IR-DIC) visualization, histochemical staining, and 3D-computer reconstruction, electrotonic coupling was recorded between close PCs, mainly in the medial prefrontal cortex as well as in the visual cortical regions of ferrets and rats. Compared with interneuron gap junctions, these electrotonic couplings were characterized by several special features. The recording probability of an electrotonic coupling between PCs is extremely low; but the junctional conductance is notably high, permitting the direct transmission of action potentials (APs) and even tonic firing between coupled neurons. AP firing is therefore perfectly synchronized between coupled PCs; Postjunctional APs and spikelets alternate following slight changes of membrane potentials; Postjunctional spikelets, especially at high frequencies, are summated and ultimately reach AP-threshold to fire. These properties of pyramidal electrotonic couplings largely fill the needs, as predicted by simulation studies, for the synchronization of a neuronal assembly. It is therefore suggested that the electrotonic coupling of PCs plays a unique role in the generation of neuronal synchronization in the neocortex.

## Introduction

Electrotonic couplings (i.e., electrical synapses or gap junctions) directly connect cytosolic contents of adjacent cells and allows direct transference of chemical and electrical signals between coupled cells. Both *in vitro* electrophysiological recordings and computer simulations demonstrate that electrical synapses play a key role in synchronizing large neuronal ensembles at different frequency bands [1], [2], [3], [4]. Neuronal synchronization has been revealed to underlie a variety of cognitive processes, such as perception, motor performance, attention, learning, and memory [1], [5]. Indeed, the significance of electrical synaptic transmission was just recently revealed to brain functions by *in vivo* patch-clamp recordings from behavior animals [6]. Electrical synaptic activity is also involved in many disorders of the central nervous system, such as epilepsy [7] and brain ischemia [8]. Owing to recent technical developments in electrophysiology, transgenic approaches, cellular imaging, as well as a high probability of inter-connection and well-defined channel proteins, electrical synapses between interneurons have been extensively studied in many cortical and sub-cortical areas with their significance to brain functions becoming more evident [1], [5], [9], [10]. However, channel proteins of electrotonic couplings amongst excitatory neurons are still not clearly identified. This uncertainty essentially restricts powerful techniques, which are commonly used for interneuron gap junctions, in the study of electrotonic couplings of excitatory pyramidal cells (PCs). These techniques include labeling gap junction proteins at synaptic sites, setting specific pharmacological blockades on coupling channels, and reducing electrical coupling following knockout/knockdown of specific genes of channel proteins. In contrast, the most practical method is the direct intracellular recording of coupled PCs, a convincing technique to demonstrate electrotonic couplings between two neurons [9], [10]. Utilizing this technique, several reports have illustrated some features of electrotonic couplings of PCs in the hippocampus and entorhinal cortex [11], [12], [13]. But only one coupled PC pair was directly recorded and reported given an extremely low coupling probability in the neocortex [13]. Despite other compelling indirect evidence, the electrophysiological, anatomical and synaptological properties of the electrical coupling of neocortical PCs are still largely unknown.

Using multi-neuron patch-clamp recording with differential interference contrast infrared videomicroscopy (IR-DIC) visualization, subsequent histochemical staining and 3D-computer reconstruction, electrotonic couplings were directly recorded from close PCs mainly in the medial prefrontal cortex (PFC) as well as in the visual cortical regions of ferrets and rats. Their features were electrophysiologically and anatomically demonstrated. In particular, postjunctional action potential (AP) firing that virtually obtained from all studied electrotonic couplings relied on the notably high junctional conductance and/or the summation of spikelets, especially at high frequencies. This postjunctional AP firing, including bursting, could be triggered at resting membrane potentials, and perfectly synchronized between coupled PCs. Several other features were also revealed. The electrotonic coupling of neocortical PCs is comprehensively characterized. This study is therefore valuable in the understanding of the role of PC electrotonic couplings in brain functions under physiological and pathological conditions.

## Results

### Coupling Probability and Anatomical Properties

**Figure 1:**
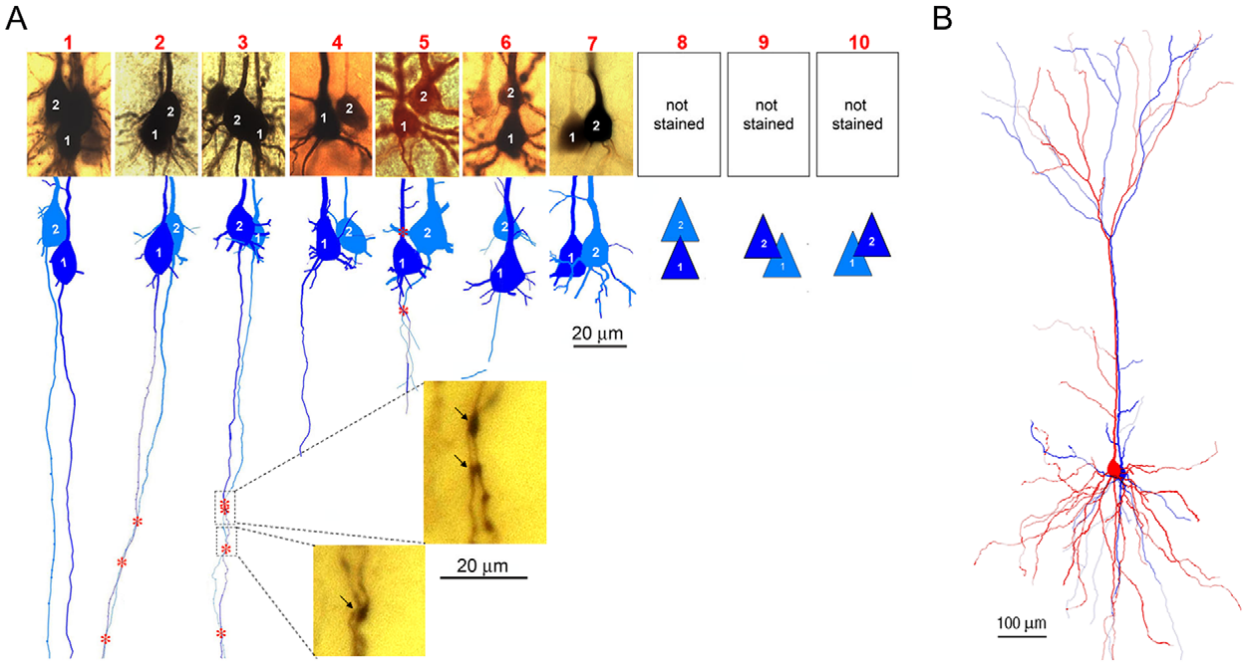
Morphologies of electrically coupled PCs in the neocortex. **A.** Images (**upper panel**) and reconstructions (**lower panel**) show the somata, primary dendrites and axonal trunks of 7 pairs. Putative contacts on dendrites and axons are marked with red asterisks on the reconstructions. The images of the coupled pairs were captured at 40× magnification and edited using the ‘merge images’ function of the Neurolucida program. Four of the pairs also had axonal trunks stained. The images of three putative axo-axonal contacts (marked with arrows in the **insets**) were captured at 60× magnification. The spatial arrangements of cells in the 3 unstained pairs are represented with schematic icons, which were derived through the visualization of recorded neurons under DIC optics during recording. In 8 of the 10 pairs, coupled PCs had touching or overlapping somata. The other 2 pairs (No. **5 &** No. **6**) had separations of only a few micrometers between somata of coupled PCs. The pair No. **5** had putative contacts on primary dendrites and axonal trunks. The pair No. **6** had one neuron's proximal apical dendrite overlapping the other cell's soma. Three (No. **2**, No. **3 &** No. **5**), out of the 4 pairs with stained axonal trunks, displayed putative axo-axonal contacts (**insets**). These contacts were within distances of 16 to 150 µm from somata. **B.** The morphologies of coupled PCs were comparable. The 3D-computer reconstruction shows whole structures of the coupled pair No. 3. Note: For more information about each pair, please see **Table S1**.

Ten electrotonic couplings were obtained by triple/quadruple patch–clamp recordings from ∼2000 layer 5 PC pairs in the medial PFC and visual cortex (VC) of ferrets and rats (**Fig. 1**, **Table S1**). These couplings included 6 in the PFC of P14 – P43 rats, 2 in the PFC of 6–7 week ferrets and 2 in the VC of 6–9 week ferrets. All were reciprocally connected. The coupling probability was 0.5% among neighboring PCs, or 5% among those with touching somata or had negligible separations (*n* = 201 pairs). This appears lower than the probability (1.4%) for the electrotonic coupling of hippocampal CA1 PCs [12], and is notably lower than the probability (>50%) for interneuron gap junctions [9], [10], [14]. Three fast-spiking (FS) interneuron gap junctions were included for comparison.

The coupled PCs were very close to each other in all of the ten studied pairs (**Fig. 1A**), which is similar to those in the hippocampus [12]. Under light microscopy, it was observed that eight of the ten pairs had touching or overlapping somata. The other two pairs had somata separated by only a few micrometers and had putative contacts either between primary dendrites or between a primary dendrite and a soma. Among 4 pairs that had both axonal trunks stained, putative axo-axonal contacts (**Fig. 1A** **inset**) were observed in 3 pairs within 16 to 150 µm from somata. The electrotonic coupling was formed predominantly between those PCs that exhibited similar electrophysiological (n = 4/4) and morphological (n = 4/5) features as identified by their firing patterns (**Fig. S1**) and morphologies (**Fig. 1B**). A similar phenomenon was also reported in interneuron gap junctions [9], [10], [14].

### High Junctional Conductance

**Figure 2:**
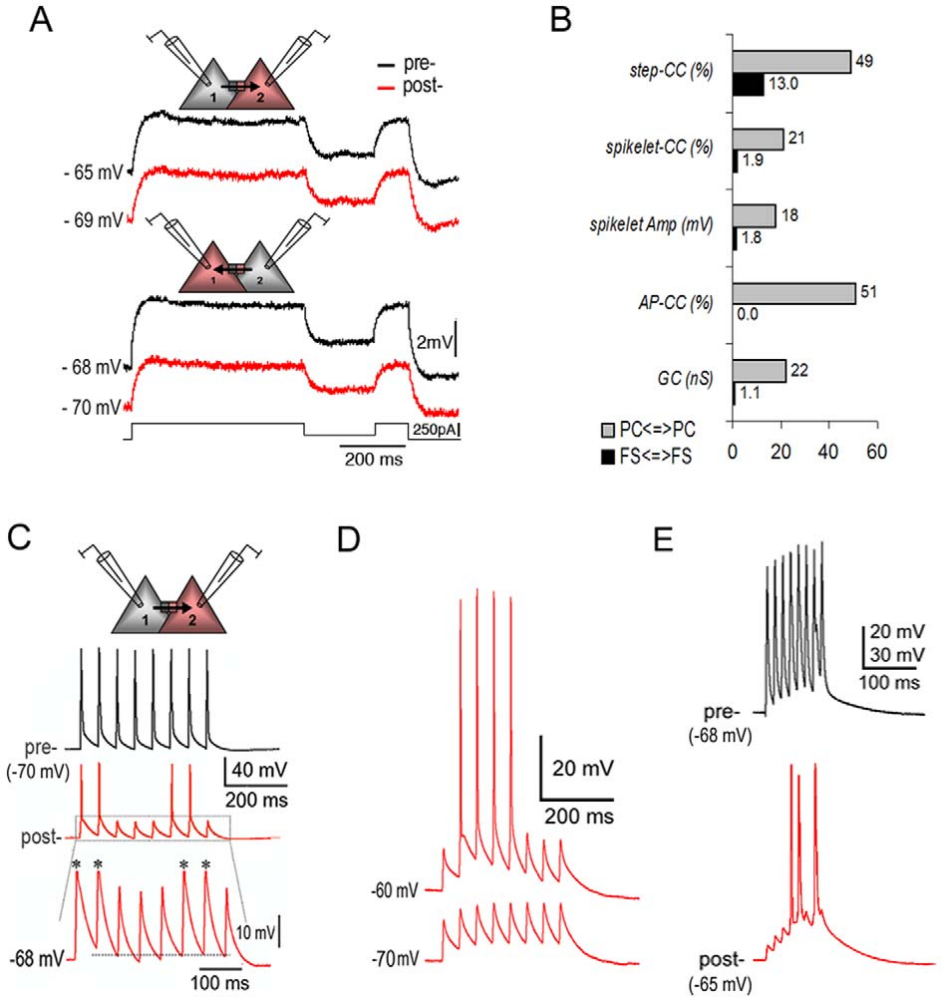
High junctional conductance and AP firing in pyramidal electrotonic couplings in the neocortex. **A.** Bi-directional sub-threshold responses to stepped-current injections to either of the coupled PCs. **B.** The histogram shows notable disparities between pyramidal and interneuron electrotonic couplings. **C.** A prejunctional AP train induced a mixture of postjunctional APs and spikelets depending upon slight variations of membrane potential levels (**inset**: A broken line indicates a ‘threshold’ for the induction of either an AP or a spikelet. Asterisks mark truncated APs). **D.** AP firing was induced due to membrane potential depolarization from a resting level of −70 mV to −60 mV. **E.** Postjunctional AP firing was generated at resting membrane potential through the summation of spikelets when a 70 Hz prejunctional AP train was triggered. Spikelet summation, to a lesser extent, was also observed at 20 Hz as shown in **D**. Note: Traces were recorded respectively from the PFC slices of a 6 week old ferret for **A**, a P18 rat for **C** and a P32 rat for **D & E**.

Electrotonic couplings were verified by recording responses of one cell (postjunctional) to sub-threshold depolarizing and/or hyperpolarizing pulses that were injected into the other cell (prejunctional) (**Fig. 2A**). The postjunctional responses were consistent in amplitudes without any failures (**Fig. S2A**). The electrotonic coupling strength was assessed by the coupling coefficient (*CC*), defined as the ratio of membrane voltage changes between the postjunctional and prejunctional cells [9]. According to properties of pre- and post-junctional responses, the *CC* was defined into 3 formats: 1) *step-CC*, when a prejunctional current-step induces a postjunctional step-response; or 2) *spikelet-CC*, when a prejunctional AP/spikelet induces a postjunctional spikelet; or 3) *AP-CC*, when a prejunctional AP induces a postjunctional AP.

The most striking feature of electrotonic couplings between PCs in the neocortex is the high *CC* **(** **Fig. 2B** **,** **Table 1** **)**. On average, the *step-CC* was more than 5-fold, the *spikelet-CC* was more than 10-fold, the spikelet amplitudes were nearly 10-fold, and junctional conductance was more than 25-times higher than those documented for interneuron gap junctions (**Table 1** **and Table S2**, also see [9]). Based on the high conductance, APs generated in a prejunctional cell resulted in APs or near AP-threshold spikelets in the postjunctional cell (**Fig. 2C**). APs and spikelets could easily alternate following slight shifting of membrane potentials. In 8 of the 10 pairs, postjunctional APs were directly triggered at resting membrane potentials by evoking prejunctional AP trains (20 Hz), which have never been observed in interneuron gap junctions [9], [10]. For the 2 pairs without APs induced at resting potentials, the postjunctional cells were still able to fire by slightly depolarizing their membrane potentials (**Fig. 2D**) or increasing prejunctional stimulation frequencies (**Fig. 2E**). In the latter case, postjunctional spikelets were summated to reach AP-threshold due to the slow decay time constant of PCs (*n* = 4/4, **Fig. S2**). To the contrary, a frequency-dependent attenuation of postjunctional responses due to a low-pass filtering property was observed in interneuron gap junctions (**Fig. S3**; also see [10], [15]).

**Table 1:** Comparison between pyramidal and interneuron electrotonic couplings.

parameter		PC < = > PC		FS < = > FS
	*n*	mean ± s.e.m. (Mini. ∼ Max.)	*n*	mean ± s.e.m. (Mini. ∼ Max.)
soma distance (µm)	10 pairs	0.7 (0∼5)**	3 pairs	13 (3, 5, 30)
reciprocal	10 pairs	10/10.	3 pairs	3/3.
postjunctional spikelet amplitude (mV)	13	14±2 (4∼28)**	6	1.5±0.20 (0.9∼2.05)
postjunctional AP amplitude (mV)	11	80±8 (45∼120)		N/A
step-coupling coefficient (*step-CC*)	9	54%±9% (20%∼93%)**	6	10.6%±2.4% (5.6%∼17.4%)
spikelet-coupling coefficient (*spikelet-CC*)	10	16%±5% (4%∼46%)**	6	1.5%±0.3% (0.8%∼2.4%)
AP-coupling coefficient (*AP-CC*)	11	53%±4% (28%∼68%)		N/A
*CC* assymetry in bidirection	10 pairs	17%±12% (3%∼42%)**	3 pairs	0.13%±0.00% (0.07%∼0.16%)
junctional conductance (GC, nS)	9	19.1±9.1 (1.9∼83.4)*	6	0.74±0.21 (0.27∼1.40)
postjunctional spikelet peak delay (ms)	13	0.80±0.12 (0.19∼1.8)	6	1.20±0.32 (0.58∼2.60)
half-duration of postjunctional spikelet (ms)	13	16±3 (4∼31)*	6	6.8±2.4 (0.2∼12.9)
half-duration of postjunctional AP (ms)	11	3.00±0.27 (1.97∼4.28)		N/A
rising time contant of postjunctional spikelet (ms)	13	1.6±0.1 (0.9∼2.0)*	6	1.1±0.2 (0.6∼1.8)
rising time contant of postjunctional AP (ms)	11	0.97±0.16 (0.56∼2.19)		N/A
decay time contant of postjunctional spikelet (ms)	13	8.20±1.10 (2.40∼13.80)*	6	14.1±2.4 (7.4∼21.6)
decay time contant of postjunctional AP (ms)	11	5.19±1.05 (1.82∼11.3)		N/A
AHP amplitude of postjunctional spikelet (mV)	13	0.56±0.18 (0.00∼2.19)	6	0.40±0.10 (0.06∼0.63)
AHP amplitude of postjunctional AP (mV)	11	1.70±0.25 (0.00∼2.81)		N/A

*p<0.05, ** *p*<0.01; N/A: No data available for postjunctional APs recorded at resting membrane potential via an interneuron gap junction.

Note 1: The *n* values can be more than 10 because electrophysiological parameters were measured in bi-directions.

Note 2: Postjunctional APs/spikelets were induced by prejunctional AP trains that were evoked by brief depolarizing currents (duration, 3 ms).

**Figure 3:**
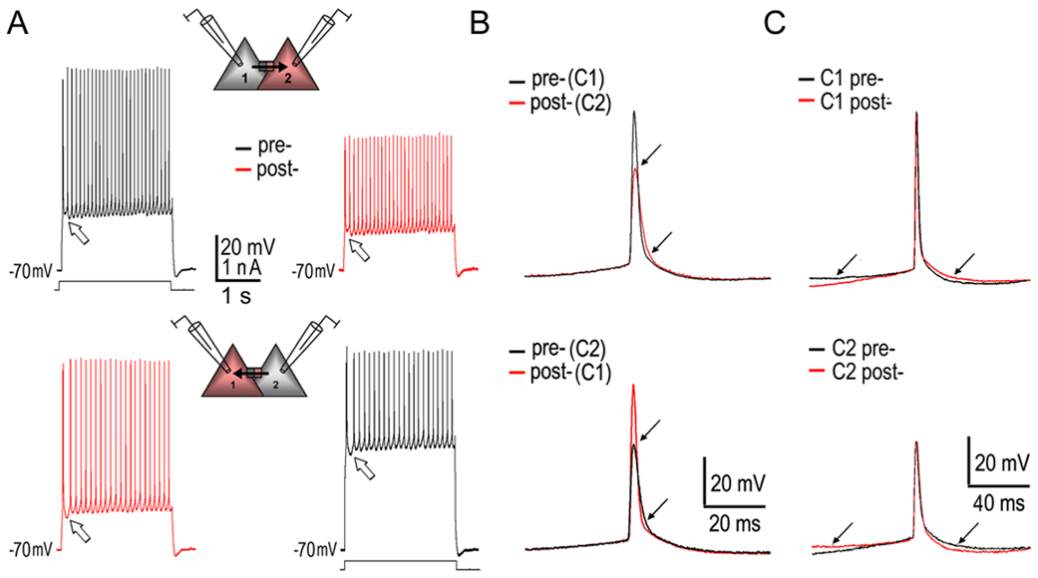
Bi-directional tonic firing via pyramidal electrotonic couplings. **A.** Tonic firing was ‘propagated’ from cell 1 to cell 2 (**upper**) and from cell 2 to cell 1 (**lower**). The firing patterns of the postjunctional cell mirrored those of the prejunctional cell. In elaboration, different initial firing patterns of cell 1 and cell 2 were exactly replicated postjunctionally (blank arrows). **B.** Corresponding to the direction of coupling conductance in **A**, the pre- (stimulating) and post-junctional (responding) APs had the identical slow rising and falling phases, but the different fast rising and falling phases (marked with arrows). **C.** The pre- and post-junctional APs of either cell (the same cells as in **A**) possessed the same fast rising and falling phases, but the different slow phases (marked with arrows). Note: AP traces in **B & C** were superimposed on each other by centering AP peaks without consideration for the onset delay of postjunctional responses. Recordings were obtained from the PFC slice of a P14 rat.

In 3 of the 10 pairs, tonic firings induced in prejunctional cells were simultaneously recorded from postjunctional cells (**Fig. 3A**). On close examination, the slow rising and falling phases of pre- and post-junctional APs perfectly overlapped when the onset delay was ignored (**Fig. 3B**). Whereas, the fast phases of postjunctional APs perfectly overlapped the postjunctional cell's own intrinsic APs (*i.e*., the APs generated by direct current injection) (**Fig. 3C**). Therefore, a postjunctional AP was a ‘hybrid’. Its slow phases resulted from a passive electrical process that was transmitted from the prejunctional AP. Its fast phases resulted from an active electrical process that was determined by its own intrinsic properties. These ‘hybrid’ APs were useful factors to exclude the possibility that two electrodes were recording from the same cell. In the latter case, APs recorded from the same cell with two electrodes overlapped perfectly on all phases of a spike (n = 5/5, **Fig. S4A**, also see [12]). Even when one electrode had input resistance increased, the APs evoked with that electrode were still perfectly congruent with those APs recorded with it but triggered by the other (n = 2/2, **Fig. S4B - right**). However, these APs could not overlap those APs recorded with the other electrode in either phase (**Fig. S4B - left**).

### Additional Features

**Figure 4:**
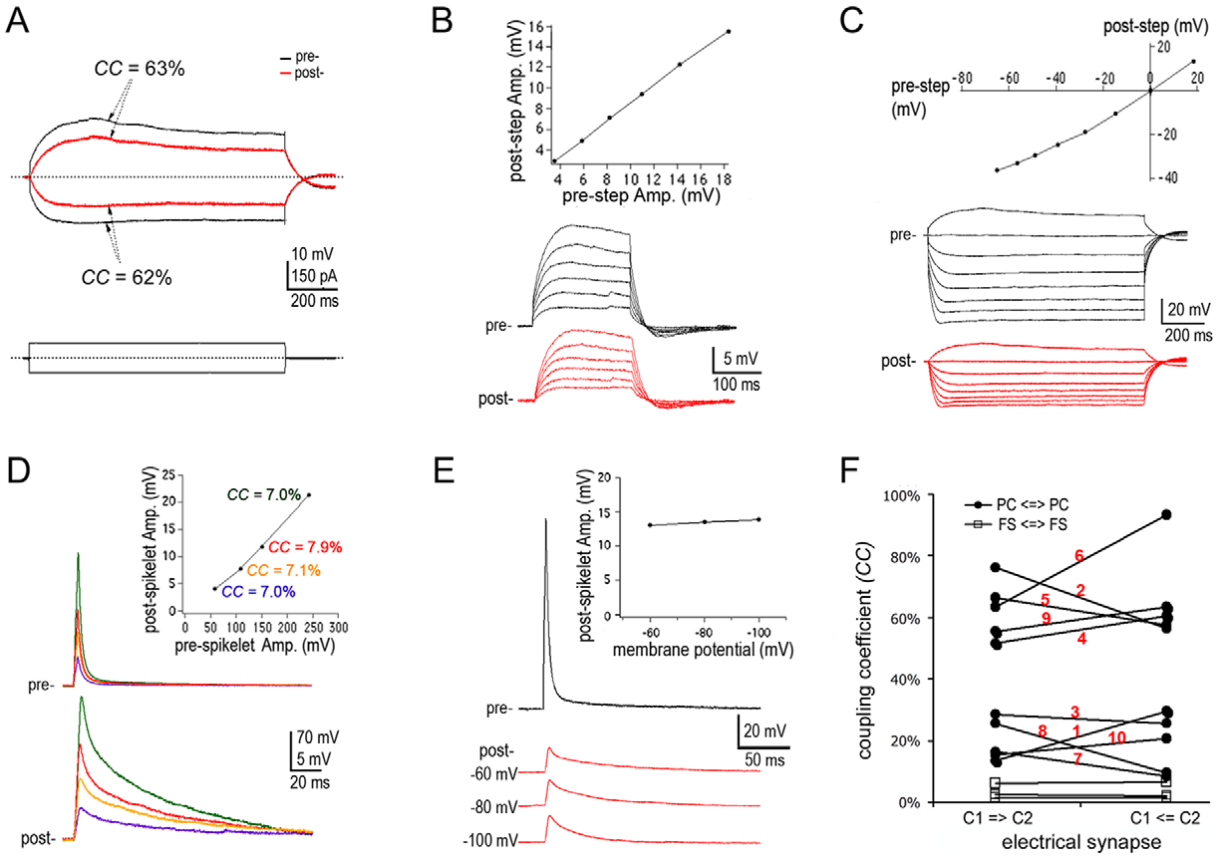
Characteristics of the electrotonic coupling between PCs in the neocortex. **A.** *CCs* are virtually symmetrical at the same current steps of depolarization and hyperpolarization. **B**. Linear relationship between pre- and post-junctional depolarizing responses. **C.** Linear relationship between pre- and post-junctional hyperpolarizing responses. **D.** Postjunctional spikelets are linearly correlated to the gradually enhancing prejunctional responses (*CCs* in the graph are color-coded with the superimposed traces of pre- and post-junctional responses). **E.** Amplitudes of spikelets induced by a pre-junctional response remained unchanged at different postjunctional membrane potentials. **F.** Comparison of asymmetrical *CCs* of pyramidal electrotonic couplings with symmetrical *CCs* of interneuron gap junctions. The *CC* label numbers correspond to the image numbers in **Fig. 1**. The *CCs* included *step-CCs* of pairs No. **2**, No. **3**, No. **6 &** No. **10** and *spikelet-CCs* or *AP-CCs* of the other pairs. Note: All responses were recorded at −70 mV except those in **E**. Traces were recorded from PFC slices of a 6 week ferret for **B** and a P32 rat for **A, C**, **D**, and **E**, respectively.

Additional features of electrotonic coupling between PCs were revealed in the neocortex. *Step-CCs* were symmetrical when the same steps of depolarizing and hyperpolarizing pulses were delivered (**Fig. 4A**, *n* = 3/3 pairs). Furthermore, a linear relationship was observed between pre- and post-junctional step responses (**Fig. 4B&C**, *n* = 6/6 pairs) as well as between prejunctional responses and postjunctional spikelets (**Fig. 4D**; *n* = 2/2 pairs). These linear relationships demonstrate the negligible voltage-dependence of channel gating in the electrotonic coupling of PCs. In accord with this property, spikelet amplitudes were constant at different postjunctional membrane potentials when a stimulation was repeated to a prejunctional cell (**Fig. 4E**; *n* = 2/2 pairs). A similar result was also observed in the electrotonic coupling of hippocampal PCs [12]. In addition, *CCs* were asymmetrical at different degrees in bi-directions, contrasting the low and symmetrical conductance of interneuron electrotonic couplings (**Fig. 4F**, see also [9], [10], [14].

## Discussion

Electrical coupling between PCs has been reported many times [16]. However, most previous studies were carried out using dye coupling that has been proven unreliable for the identification of electrotonic couplings (see review [10]). This may be because coupling channel proteins, such as connexin and pannexin, form hemichannels that can directly take up dyes from the interstitium [17], [18]. The most convincing methods include the observation of ultrastructures of gap junctions under electron microscopy (EM) and direct intracellular recording of coupled neurons [9], [10]. Spines, a structure specialized for excitatory neurons, were found to form gap junctions with dendrites and somata in the cortex in early EM studies [19], [20]. Given the low coupling probability of pyramidal gap junctions and technical difficulties, EM studies are rare and results are often unsatisfying [12]. Using direct intracellular recordings, electrotonic couplings of PCs have been demonstrated in the hippocampus and entorhinal cortex by a few research groups [11], [12], [13]. Yet in the neocortex, only one coupled PC pair was directly recorded and reported in a recent study using sharp electrode intracellular recording [12]. Moreover, this study has provided additional evidence using an indirect intracellular pair recording technique. In the recording of a chemical synaptic connection, spikelets of the presynaptic cell appear to elicit EPSPs (excitatory postsynaptic potentials) in the postsynaptic cell. In fact, the ‘spikelets’ are a direct result of APs evoked in a 3rd unrecorded neuron that is electrically coupled to the presynaptic cell and simultaneously forms chemical synapses on the same postsynaptic cell. Spikelets recorded from PCs were also recently observed in behavior animals [6]. Using *in vivo* intracellular patch-clamp recording, spikelets of PCs were found to strongly contribute to spiking activity during spatial exploration by freely moving rats. This delicate study reveals an indispensable role of electrical synaptic transmission via PCs in brain functions. In the current study, a significantly greater number of coupled PC pairs were directly recorded mainly from the PFC using patch-clamp recordings. The electrotonic coupling of neocortical PCs is described in terms of synaptology, electrophysiology and morphology.

### Special Electrical Synaptic Dynamics

Electrotonic couplings between PCs are considered essential for ultrafast frequency synchronizations (100–600 Hz) in hippocampal and neocortical areas [1], [21], [22], [23], [24], [25] while interneuron gap junctions are closely related to fast frequency synchronizations (4–12 Hz for θ and 20–70 Hz for γ frequencies) [9], [10], [26]. In enabling ultrafast frequency synchronizations, electrotonic couplings of PCs should have high conductance and allow direct AP propagation according to simulation studies [24], [27]. Our work provides experimental evidence supporting this theory. AP firing, even bursting, triggered in a prejunctional cell is directly ‘propagated’ into a postjunctional cell. This phenomenon has been observed in previous studies as well [11], [12], [13]. This property leads to a sub-millisecond spike coordination between PCs, representing an extraordinary ability for neuronal synchronization and signal amplification. However, it could be speculated that the high conductance coupling was artificially formed by membrane fusion owing to damages from cutting and/or recording procedures. In our experiments, neurons that were 20∼60 µm below the cutting surface of a slice were chosen for recording in order to avoid the cutting-damage on somata and proximal dendrites and axonal trunks. Recording pipettes approached and patched onto two neurons under direct DIC visualization. The patching procedure, which employed gentle suction to the soma membrane with the pipette tip, appears unlikely to make the two cells ‘penetrated’. If sharp electrodes are used, the penetration of two cells may occur and result in the membrane fusion forming an artificial electrotonic coupling.

Another property relevant to the high conductance is the easy alternation between postjunctional APs and spikelets, which is dependent upon slight changes of membrane potential levels as well as AP-thresholds. This influence of intrinsic membrane properties on the active state of a coupled cell will further extend to the electrical network, and ultimately to the whole neuronal network. This finding supports a known theory - that interactions between electrical synaptic and intrinsic membrane properties essentially result in neuronal synchronous activity in electrical networks [28],[29],[30].

Electrotonic coupling of PCs in the neocortex displays additional features. Postjunctional spikelets summate and eventually attain AP-threshold firing due to the slow decay time constant of PCs and the high conductance of electrotonic couplings. The summation is more apparent at high frequencies. To the contrary, APs are ‘filtered’ by interneuron gap junctions while relatively small and slow signals such as subthreshold activities are communicated more effectively (see review [9], [10]). This property named low-pass electrical filtering determines that interneuron gap junctions exhibit a frequency-dependent attenuation in postjunctional responses [10], [15]. EPSPs of chemical synaptic transmissions also become depressed at high frequencies, which is determinant on insufficient transmitter release in subsequent synaptic responses [10], [15], [31], [32]. In view of the fact that strengths of both interneuron gap junctions and excitatory chemical synapses are becoming weakened, the summation property might essentially make pyramidal electrotonic couplings available for ultrafast frequency synchronizations [23], [24], [25].

Previous studies have predicted that channels of pyramidal electrotonic coupling may be significantly different from those of interneuron gap junctions. In the transgenic mice with interneuron gap junction protein knockout, ultrafast frequency oscillations that depend on pyramidal electrical synaptic transmission remain intact and are sensitive to gap junction blockers [21], [22], [23]. Pannexins show a significantly high conductance [17], [33] and a remarkably low voltage sensitivity [34]. These characteristics are consistent with the observations of the current study. This supports the assumption that pannexins are the channel proteins coupling PCs [21], [22], [23].

Furthermore, asymmetrical bi-directional conductance was exhibited in all studied PC pairs. The difference in bi-directional *CC*s can easily result from the imbalanced input resistances between recording pipettes, the differential intrinsic membrane resistances and unhealthy conditions between coupled PCs. However, it cannot completely exclude the possibility of a special property for pyramidal electrotonic couplings. The asymmetrical property has already been reported in an early direct recording of electrical synapses in grayfish [35]. Because invertebrate animal gap junction proteins, innexins, share structural features with pannexins (see review [1]), it is not surprising to see the asymmetrical conductance in the pyramidal electrotonic couplings that are most possibly formed by pannexins. Indeed, the asymmetrical *CC*s were also obtained from reliable recordings of identical healthy PCs with balanced electrodes (such as the one in figure 2A), but were not apparent in those of interneuron gap junctions (see Fig. 4F and also [9], [10]).

### Sparse Distribution

The probability of recording an electrotonic coupling between PCs is extremely low in the neocortex. It is reasonable to question how pyramidal electrical synapses contribute to the synchrony of a neuronal network. First, the high conductance is crucial [7], [24]. Simulating pyramidal neuronal networks in the absence of chemical synaptic signaling demonstrated that the high conductance, specifically the direct AP propagation between neurons, enables an immediate and fast signal transference over long distances. This feature determines that a sparse distribution is necessary for pyramidal electrical synaptic networks to function properly. Otherwise, epileptogenesis may be induced [7], [25]. Both the high conductance and low coupling probability are confirmed in the current study. The junctional conductance of pyramidal electrotonic couplings in the neocortex appears to be even greater than that in the hippocampus [12] (*CC*: 54% vs. 25%). This could better explain a sparser distribution (coupling probability: 0.5% in the neocortex vs.1∶72 or 1.4% in the hippocampus ). Second, the opening of pyramidal electrotonic coupling channels may normally be restricted for some unknown reasons. This possibility is indicated by the fact that pyramidal electrical synaptic activity and neuronal synchronization are significantly enhanced under certain conditions, such as ischemia, low-calcium or calcium-free conditions [8], [24]. Finally, electrotonic coupling between PCs may be unevenly distributed in the neocortex. The PFC possesses special features in the organization of chemical synaptic networks [31]. It may also be specialized in the organization of electrical synaptic networks, such as in maintaining a higher number of pyramidal gap junctions for higher degrees of synaptic activity and plasticity. In light of these findings in the PFC, it would be interesting to explore other associative cortices. By selectively recording small tight clusters of PCs, the probability of recording an electrotonic coupling can be increased to a rate comparable to those of chemical synapses in some primary cortical areas [36], [37], [38]. This selective method makes direct recording of the rare PC couplings feasible. Interestingly, the clustering construct of electrically coupled excitatory neurons has also been predicted in an earlier modeling study [25].

### Proximal Coupling Sites

Electrical synaptic contacts could be formed at multiple central cellular compartments including somatic, and proximal dendritic and axonal sites according to previous studies on gap junctions of excitatory neurons [39], [40], [41]. Consistent with this, all the coupled PCs were assembled together with either overlapping somata or had negligible separations. This anatomical feature, as well as the high conductance, implies that electrical synaptic contacts are most likely formed on somata and proximal dendritic and axonal structures. Axo-axonal contacts have been emphasized for the pyramidal electrotonic coupling [7], [39]. We also found putative axo-axonal contacts in three out of the four pairs that had stained axonal trunks. However, responses recorded from these pairs cannot be fully explained by axo-axonal contacts alone. For instance, the electrotonic coupling No. 2 in figure 1 had putative axo-axonal contacts approximately 150 µm away from somata. Through such a distance to soma, a subthreshold electrical signal will be attenuated by more than 80% (personal communication with Dr. Y. Shu). Whereas, the bi-directional *step-CCs* were well over 20% (cell 1 to cell 2: 76% vs. cell 2 to cell 1: 56%). These high *step-CCs* would be better explained if proximal contacts were also taken into consideration.

### Summary

According to electrical network simulations, pyramidal gap junctions require special features for the synchronization of a large assembly of cortical neurons [7], [24]. The current study verifies that these features indeed exist in the neocortex, which include: the low coupling probability, making pyramidal electrical synaptic networks necessarily sparse; the high conductance, allowing direct propagation of full APs from neuron to neuron; the easy alternation between postjunctional APs and spikelets following slight changes of membrane potentials and AP-thresholds, serving as a switchmodulator for neuronal networks; the summation of postjunctional spikelets, making pyramidal electrotonic couplings reliable for ultrafast frequency synchronizations. In addition, the asymmetrical bi-directional conductance may enable preferential transference for some signals. The electrotonic coupling between PCs is the only synaptic type possessing these special features. Therefore, it may serve as a fundamental synaptic apparatus for generating neuronal synchronizations in the neocortex, and hence could be important to many relevant physiological and pathological states.

## Materials and Methods

### Ethics Statement

Ferrets and Wistar rats were used acutely for the purpose of obtaining and preparing brain slices. All the research involving animals have been approved by the Tufts University Institutional Animal Care and Use Committee. Housing and surgical procedures of animals used for recording were in accordance with the National Institutes of Health guidelines and the Tufts University Institutional Animal Care and Use Committee.

### Brain Slice Preparation

Young ferrets (6–9 weeks) and rats (14–43 days) were anesthetized by using IP injection of sodium pentobarbital (200 mg/kg), and decapitated, and their brains were immediately removed and placed in cold artificial cerebrospinal fluid (ACSF). Horizontal or coronal slices (300 µm) were sectioned from the medial PFC by using a vibratome (DTK 1000 Zero 1, Microslicer, Japan). Horizontal slices were prepared from ferrets, coronal slices from rats. The cutting angle was always adjusted in order to make the main axis of neurons parallel to the cutting surface. For this purpose, a parallel cortical blood vessel was a reliable referring landmark. Brain sections were transferred into ACSF which was continuously oxygenated with 95% O_2_ and 5% CO_2_, incubated for 30 min at 34 degrees (°C) and then at room temperature (20–22°C) until transferred to the recording chamber. During recording, brain slices were maintained at 34°C in a recording chamber and perfused with oxygenated ACSF at a flow rate of 0.75–1.0 ml/min. The ACSF solution contained (mM): 125 NaCl, 2.5 KCl, 25 glucose, 25 NaHCO3, 1.25 NaH2PO4, 2 CaCl2, and 1 MgCl2. Neurons in layer 5 were identified using IR-DIC optics, with an upright microscope (BX50WI, Olympus, fitted with 40x-W/0.8 NA objective, Olympus, Japan) in accordance to the pyramidal soma shape and thick primary apical dendrites typical for PCs. Recorded PCs were further verified through observation of stained neurons under light microscopy and 3D-computer reconstructions.

### Electrophysiological Recording

Triple/quadruple patch-clamp recordings were carried out to capture electrical synaptic connections formed between single neurons that were approximately 20–60 µm under the cutting surface of a slices. Somatic whole-cell recordings (pipette resistance 6∼12 mΩ) were made, in which access resistance was determined from settings of bridge balance in experiments where Axoclamp-200B amplifiers (Molecular Devices, Sunnyvale, CA, USA) were used. Series resistance compensation was enabled (90% correction) and monitored throughout the recordings. Signals were amplified using Axoclamp-200B amplifiers and collected only when the series resistance was relatively stable (changes were less than 30% of original). Recordings were sampled at intervals of 10–400 µs and filtered at 3, 10 or 30 kHz using Pulse Control (InstruTECH, Great Neck, NY, USA) and program Igor (Igor Wavemetrics, Lake Oswego, OR, USA), digitized by an ITC-18 interface (InstruTECH) and stored on hard drive (Macintosh G4 computer) for off-line analysis (Igor). Voltages were recorded with pipettes containing (mM): 100 potassium gluconate, 20 KCl, 4 ATP-Mg, 10 phosphocreatine, 0.3 GTP, 10 Hepes (pH 7.3) and 0.4% biocytin (Sigma). The recorded neurons were filled with biocytin by diffusion through a recording pipette.

### Data Analysis for Gap Junctions

Prejunctional cells were stimulated with two kinds of current injections, the step currents (300 ms to 1 s duration) and a train of eight stimulating pulses at 20 to 70 Hz (3 ms duration per pulse). A full or partial postjunctional AP was defined in accordance with a postjunctional response exceeding AP-threshold. A spikelet was defined in accordance with a subthreshold postjunctional response evoked by a prejunctional spikelet or an AP. The step coupling-coefficient, *Step-CC*, was calculated as the ratio of postjunctional to prejunctional step-voltages. *Spikelet-CC* was the amplitude ratio of postjunctional spikelets to prejunctional spikelets or prejunctional APs. *AP-CC* was the amplitude ratio of postjunctional APs to prejunctional APs. Assuming a model of two isopotential neurons and a single junction, gap junctional conductance was determined (*GC*)  =  1/[(*R_in_*/*CC*)-*R_in_*] (*R_in_*: input resistance of the postjunctional cell, *CC*: *step-CC*) [9], [42], [43]. Input resistances were approximated by linear regression of voltage deflections. Spikelets/AP amplitudes were determined by average peak values (5–10 values/peak). The postjunctional peak delay was the difference between pre- and post-spikelets/AP peak times. The coupling-coefficient asymmetry is the difference of *CCs* in bi-directions. The half-duration of a postjunctional spikelet/AP was measured at the half amplitude of a spikelet/AP. The rise time constant was the time to rise from 20% to 80% peak amplitudes of a postjunctional spikelet/AP. The decay time constant was the time to decay to 80% of the peak amplitude of a postjunctional spikelet/AP. The electrotonic couplings in the current study were mostly secondary results of the recordings for chemical synaptic transmissions. Pharmacological characterization of electrical synapses was not performed.

### Histological Procedures and 3D Computer Reconstruction

After recording, slices bearing biocytin-injected neurons were fixed for at least 24 hours in cold 0.1 M phosphate buffer (PB, pH 7.4) containing 2% paraformaldehyde, 1% glutaraldehyde and 0.3% saturated picric acid. Thereafter, slices were rinsed several times (10 min each) in PB. To block endogenous peroxidases, slices were transferred into phosphate-buffered 3% H_2_O_2_ for 30 minutes. After five to six rinses in PB (10 min each), slices were incubated overnight at 4°C in avidin-biotinylated horseradish peroxidase according to a manufacturer's protocol (ABC-Elite, Vector Labs, Petersborough, UK) (2% A, 2% B and 1% Triton-100). Following incubation, sections were rinsed several times again in PB and developed with diaminobenzidine (DAB) under visual control using a bright-field microscope (Zeiss Axioskop) until all processes of cells appeared clearly visible (usually after 2–4 min). The reaction was stopped by transferring sections into PB. After a rinse in the same buffer, slices were mounted onto glass slides in an aqueous mounting medium.

3D neuron models were reconstructed using the Neurolucida system (MBF Bioscience, USA) and a bright-field light microscope (Olympus, BX51, Japan). Putative electrical synapses were identified as the dendritic or axonal contacts at the same focusing plane using a microscope water lens (60× magnification, numerical aperture  = 0.9; resolution along the Z-axis  = 0.37 µm). Considering the notably high conductance of pyramidal electrotonic couplings, putative contacts on somata, primary dendrites and axonal trunks attracted special attention.

### Statistical Analysis

Student *t-test* was used for the comparison between two groups of data. *X^2^* test was used for the comparison between tow rates. Statistical significance was determined by P≤0.05.

## Supporting Information

Table S1 Information of the electrotonically coupled pyramidal pairs in the neocortex.
(0.03 MB DOC)

Table S2 Comparison of electrical synapses formed between pyramidal cells and between interneurons in the neocortex.
(0.26 MB DOC)

Figure S1 AP firing patterns of electrotonically coupled PCs were identical. The non-adapting AP firings were generated by a direct current injection into two PCs respectively. These electrotonically coupled PCs were recorded from a PFC slice of a 6-week ferret (also see Fig. 1A - No. 9 pair, and Fig. 2A for coupling responses).
(0.04 MB DOC)

Figure S2 The summation of postjunctional spikelets. A. The measurement of spikelet summating rate. This graph was generated by a single trace from a set of 20 equivalent traces. No failures were observed across all spikelet trains of the 20 traces. B. No correlation between the coupling coefficient and spikelet summation. By giving a prejunctional stimulation train at 20 Hz, the summation of postjunctional spikelets varied vastly from 0% to 56% (mean ± SE: 14%±5%; n = 6. In the other 4 pairs, the 1st and/or 2nd postjunctional responses during the train were APs in one or bi-directions.). This variation is determinant on the decay time constant of coupled PCs rather than the coupling coefficient. C. The correlation between stimulation frequency and spikelet summation. Out of the ten electrotonically coupled pairs, two of them were recorded at different stimulation frequencies. The bidirectional CCs were color-coded with grey and black for the two pairs respectively. The summation of postjunctional spikelets became strengthened while the prejunctional stimulation frequency was increased. The 2nd spikelets were summated by up to 115% of 1st spikelets at 70 Hz.
(0.21 MB DOC)

Figure S3 Postjunctional responses of a FS interneuron gap junction were increasing as the intensity of prejunctional APs became gradually reduced. The step-CC of this interneuron gap junction was 16%.
(0.06 MB DOC)

Figure S4 APs of one neuron recorded with two pipettes. A. APs recorded with the two pipette electrodes (e1 and e2) perfectly overlap each other in all phases when being stimulated with either electrode. Traces of e1 are in red, and traces of e2 are in black. B. When the impedance of the e2 electrode was notably increased afterwards, APs recorded with the two electrodes could not overlap in either phase (left panel). Whereas the APs evoked with the e2 electrode (black trace) still perfectly overlapped those APs recorded with the same electrode but evoked with e1 electrode (blue trace) (right panel).
(0.18 MB DOC)
