# Supplementary figures and images for: Electrotonic Coupling between Pyramidal Neurons in the Neocortex
Source: PLoS One. 2010 Apr 26;5(4):e10253. doi: 10.1371/journal.pone.0010253 (PMC2859939; doi:10.1371/journal.pone.0010253)

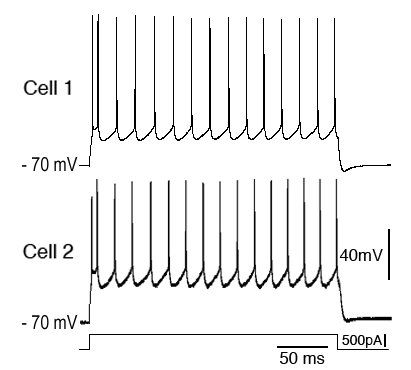

Supplement: Figure S1 — AP firing patterns of electrotonically coupled PCs were identical. The non-adapting AP firings were generated by a direct current injection into two PCs respectively. These electrotonically coupled PCs were recorded from a PFC slice of a 6-week ferret (also see Fig. 1A - No. 9 pair, and Fig. 2A for coupling responses). (0.04 MB DOC) [file pone.0010253.s003.doc]

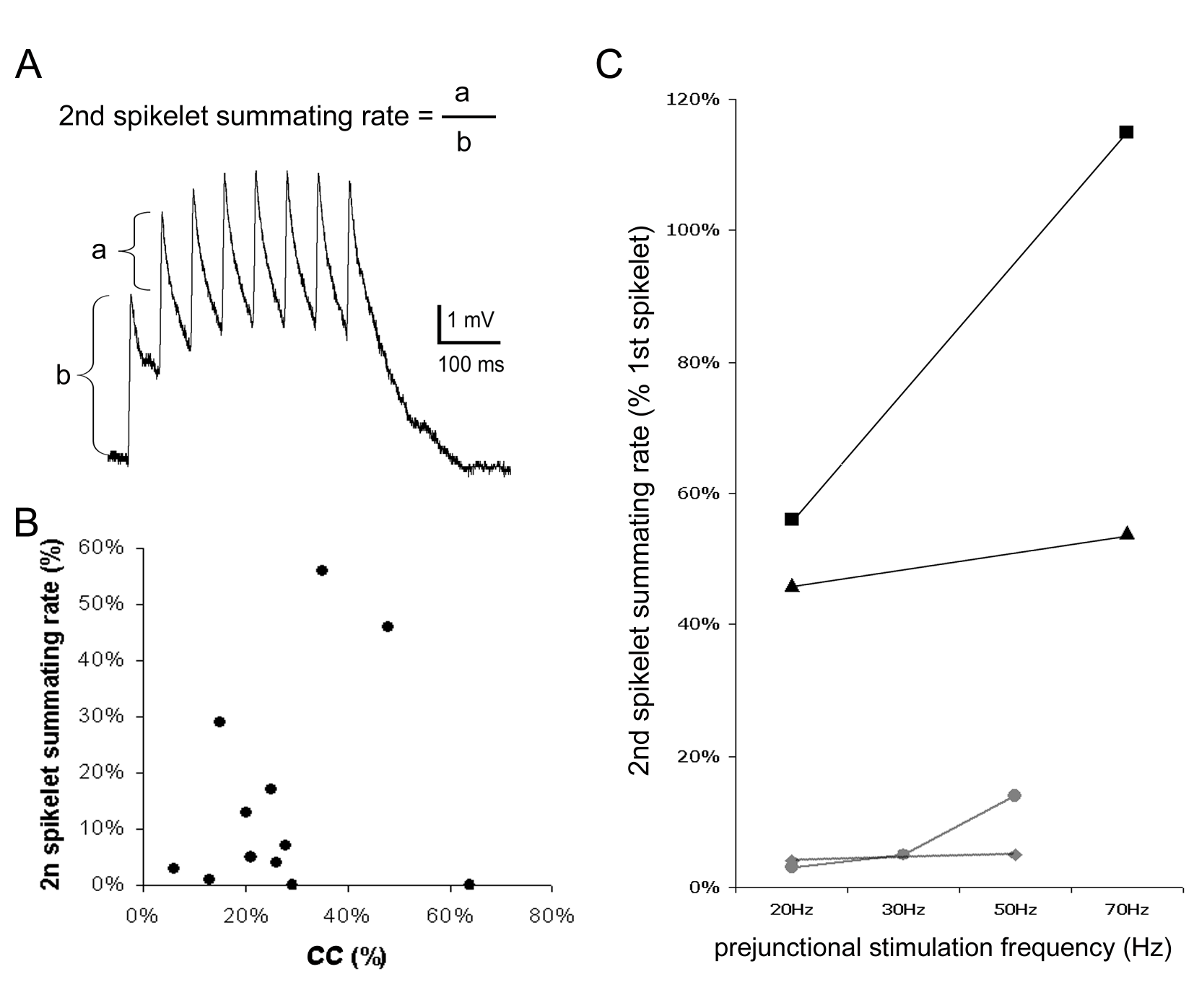

Supplement: Figure S2 — The summation of postjunctional spikelets. A. The measurement of spikelet summating rate. This graph was generated by a single trace from a set of 20 equivalent traces. No failures were observed across all spikelet trains of the 20 traces. B. No correlation between the coupling coefficient and spikelet summation. By giving a prejunctional stimulation train at 20 Hz, the summation of postjunctional spikelets varied vastly from 0% to 56% (mean ± SE: 14%±5%; n = 6. In the other 4 pairs, the 1st and/or 2nd postjunctional responses during the train were APs in one or bi-directions.). This variation is determinant on the decay time constant of coupled PCs rather than the coupling coefficient. C. The correlation between stimulation frequency and spikelet summation. Out of the ten electrotonically coupled pairs, two of them were recorded at different stimulation frequencies. The bidirectional CCs were color-coded with grey and black for the two pairs respectively. The summation of postjunctional spikelets became strengthened while the prejunctional stimulation frequency was increased. The 2nd spikelets were summated by up to 115% of 1st spikelets at 70 Hz. (0.21 MB DOC) [file pone.0010253.s004.doc]

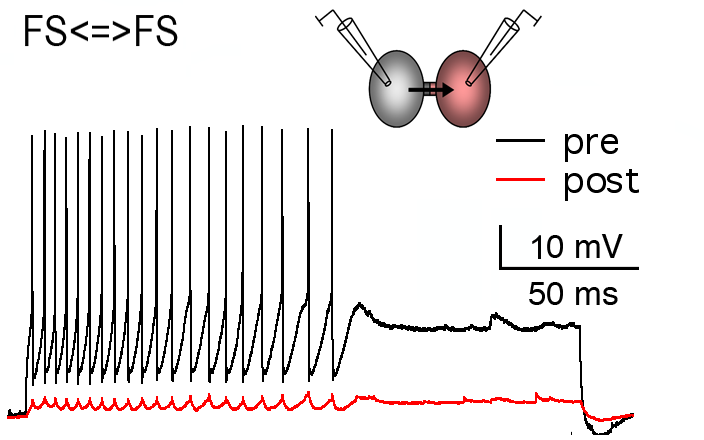

Supplement: Figure S3 — Postjunctional responses of a FS interneuron gap junction were increasing as the intensity of prejunctional APs became gradually reduced. The step-CC of this interneuron gap junction was 16%. (0.06 MB DOC) [file pone.0010253.s005.doc]

**
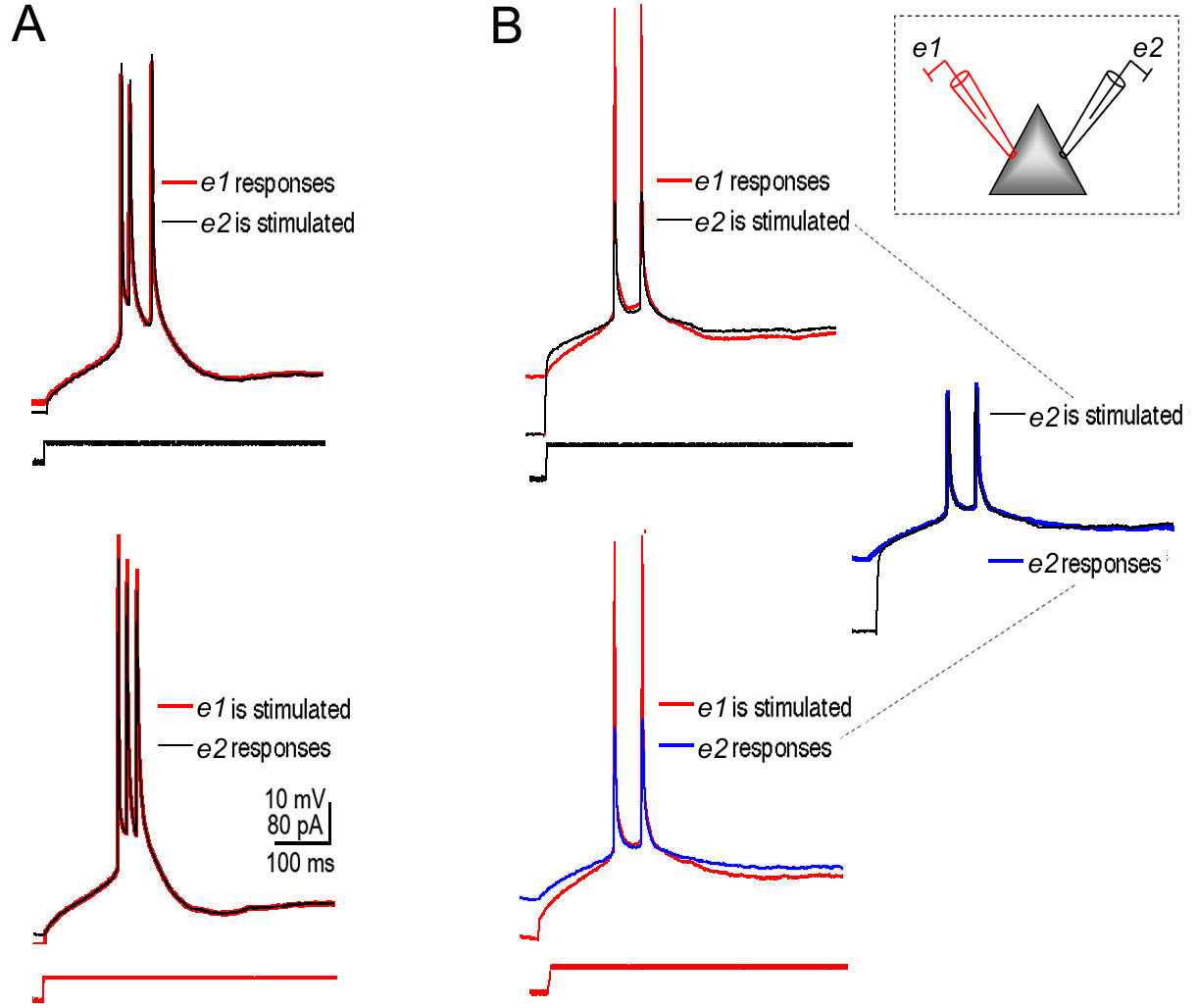
**

Supplement: Figure S4 — APs of one neuron recorded with two pipettes. A. APs recorded with the two pipette electrodes (e1 and e2) perfectly overlap each other in all phases when being stimulated with either electrode. Traces of e1 are in red, and traces of e2 are in black. B. When the impedance of the e2 electrode was notably increased afterwards, APs recorded with the two electrodes could not overlap in either phase (left panel). Whereas the APs evoked with the e2 electrode (black trace) still perfectly overlapped those APs recorded with the same electrode but evoked with e1 electrode (blue trace) (right panel). (0.18 MB DOC) [file pone.0010253.s006.doc]
