# Supplementary material for: Electrotonic Coupling between Pyramidal Neurons in the Neocortex
Source: PLoS One. 2010 Apr 26;5(4):e10253. doi: 10.1371/journal.pone.0010253 (PMC2859939; doi:10.1371/journal.pone.0010253)
Supplement: Supplementary file 2 [file pone.0010253.s001.doc]

Table S1. Information of the electrotonically coupled pyramidal pairs in the neocortex										
order in Fig.1A and Fig.4F	1	2	3	4	5	6	7	8	9	10
age	P14	P14	P18	P27	8.5w	6w	P32	7w	6w	P43
species/area	rat PFC	rat PFC	rat PFC	rat PFC	ferret VC	ferret VC	rat PFC	ferret PFC	ferret PFC	rat PFC
contribution to figure		Fig.3A,B,C	Fig.1B;Fig.2C				Fig.2D,E;Fig.4A,C,D,E		Fig.2A;Fig.4B;Fig.S1	

Note: Fig. S2 were made with the data of pairs No.1, 3, 4, 6, 9 & 10.
