# Supplementary material for: Electrotonic Coupling between Pyramidal Neurons in the Neocortex
Source: PLoS One. 2010 Apr 26;5(4):e10253. doi: 10.1371/journal.pone.0010253 (PMC2859939; doi:10.1371/journal.pone.0010253)
Supplement: Supplementary file 3 [file pone.0010253.s002.doc]

Table S2. Comparison of electrical synapses formed between pyramidal cells and between interneurons in the neocortex

	species/cortics [1]	age	cell type [2]	*n*	mean ± s.e.m.	range	reference [3]
**connecting probability**	ferret&rat/PFC&VC	14d-2m	**PC=PC**	2000	**0.5%**		current study
	ferret&rat/PFC&VC	14d-2m	**PC=PC**	20 *	**5.0%**		current study
	rats/SS&VC	14-18d	FS=FS	29	66%		Galarreta, 1999 [1]
	mice/neocortex	2-7m	FS=FS	14	61%		Galarreta, 2002 [2]
	mice /SS	juvenile	FS=FS	12	57%		Deans, 2001 [3]
	mice /SS	juvenile	LTS=LTS	3	60%		Deans, 2001 [3]
	rat/SS	20-35d	NGF=NGF	8	50%		Simon, 2005 [4]
	rat/SS	20-35d	NGF=FS	6	19%		Simon, 2005 [4]
	rat/SS	20-35d	NGF=RS	6	20%		Simon, 2005 [4]
	rat/SS	14-21d	FS=FS	24	62%		Gibson, 1999 [5]
	rat/SS	14-21d	LTS=LTS	11	85%		Gibson, 1999 [5]
	rat/SS	14-21d	FS=LTS	3	9.4%		Gibson, 1999 [5]
**soma distance (µm)**	ferret&rat/PFC&VC	14d-2m	**PC=PC**	10 pairs	**0.7**	0 - 5	current study
	ferret&rat/PFC&VC	18d, 50d	FS=FS	3 pairs	13	3 - 30	current study
	rats/SS&VC	14-18d	FS=FS	29		15 - 270	Galarreta, 1999 [1]
	mice/neocortex	2-7m	FS=FS	23	39 ± 4.2	13 - 92	Galarreta, 2002 [2]
	rat/SS	20-35d	NGF=NGF	8	39 ± 13		Simon, 2005 [4]
	rat/SS	20-35d	NGF=FS	6	31 ± 16		Simon, 2005 [4]
	rat/SS	20-35d	NGF=RS	6	28 ± 19		Simon, 2005 [4]
**step-coupling coefficient (*step-CC*)**	ferret&rat/PFC&VC	14d-2m	**PC=PC**	9	**54%± 9%**	**20% - 93%**	current study
	ferret&rat/PFC&VC	18d, 50d	FS=FS	6	10.6%± 2.4%	5.6% - 17.4%	current study
	rats/SS&VC	14-18d	FS=FS	29	6.4%± 1.7%	0.3% - 41.1%	Galarreta, 1999 [1]
	mice/neocortex	2-7m	FS=FS	14	1.53%± 0.34%	0.1% - 4.9%	Galarreta, 2002 [2]
	rat/SS	20-35d	NGF=NGF	8	1.89%± 0.77%		Simon, 2005 [4]
	rat/SS	20-35d	NGF=FS	6	3.70%± 1.42%		Simon, 2005 [4]
	rat/SS	20-35d	NGF=RS	6	4.59%± 3.98%		Simon, 2005 [4]
	rat/SS	14-21d	FS=FS	24	7%± 6%		Gibson, 1999 [5]
	rat/SS	14-21d	LTS=LTS	11	10%± 9%		Gibson, 1999 [5]
	mice /SS	juvenile	FS=FS	12	6.1%± 5.4%		Deans, 2001 [3]
	mice /SS	juvenile	LTS=LTS	3	9.6%± 4.5%		Deans, 2001 [3]
**spikelet-coupling coefficient (*spikelet-CC*)**	ferret&rat/PFC&VC	14d-2m	**PC=PC**	10	**16%± 5%**	**4% - 46%**	current study
	ferret&rat/PFC&VC	18d, 50d	FS=FS	6	1.5%± 0.3%	0.8% - 2.4%	current study
	mice/neocortex	2-7m	FS=FS	9	0.51%± 0.10%		Galarreta, 2002 [2]
	rat/SS	20-35d	NGF=NGF	8	0.17%± 0.06%		Simon, 2005 [4]
	rat/SS	20-35d	NGF=FS	6	0.39%± 0.23%		Simon, 2005 [4]
	rat/SS	20-35d	NGF=RS	6	1.01%± 0.60%		Simon, 2005 [4]
	rat/SS	14-21d	FS=FS	14	1.0%± 0.6%		Gibson, 1999 [5]
	rat/SS	14-21d	LTS=LTS	7	1.5%± 1.0%		Gibson, 1999 [5]
**AP-coupling coefficient (*AP-CC*)**	ferret&rat/PFC&VC	14d-2m	**PC=PC**	11	**53%± 4%**	**28% - 68%**	current study
**junctional conductance (*GC*, nS)**	ferret&rat/PFC&VC	14d-2m	**PC=PC**	9	**19.1 ± 9.1**	**1.9 - 83.4**	current study
	ferret&rat/PFC&VC	18d, 50d	FS=FS	6	0.74 ± 0.21	0.27 - 1.40	current study
	rats/SS&VC	14-18d	FS=FS	20	0.66 ± 0.18	0.03 - 3.30	Galarreta, 1999 [1]
	mice/neocortex	2-7m	FS=FS	14	0.203 ± 0.044	0.013 - 0.586	Galarreta, 2002 [2]
	rat/SS	14-21d	FS=FS	24	1.6 ± 1.3	0.4 - 5.5	Gibson, 1999 [5]
	rat/SS	14-21d	LTS=LTS	11	2.1 ± 2.3	0.3 - 6.9	Gibson, 1999 [5]
***CC* asymmetry in bi-directions**	ferret&rat/PFC&VC	14d-2m	**PC=PC**	10 pairs	**17%± 12%**	**3% - 42%**	current study
	ferret&rat/PFC&VC	18d, 50d	FS=FS	3 pairs	0.13%± 0.00%	0.07%, 0.15%, 0.16%	current study
**postjunctional spikelet amplitude (mV)**	ferret&rat/PFC&VC	14d-2m	**PC=PC**	9	**14 ± 2**	**4 - 28**	current study
	ferret&rat/PFC&VC	18d, 50d	FS=FS	6	1.5 ± 0.20	0.9 - 2.05	current study
	rat/SS	20-35d	NGF=NGF	8	0.62 ± 0.79	0.09 - 2.08	Simon, 2005 [4]
	rat/SS	20-35d	NGF=FS	6	0.52 ± 0.23	0.17 - 0.87	Simon, 2005 [4]
	rat/SS	20-35d	NGF=RS	6	0.45 ± 0.38	0.18 - 0.72	Simon, 2005 [4]
**postjunctional AP amplitude (mV)**	ferret&rat/PFC&VC	14d-2m	**PC=PC**	11	**80 ± 8**	**45 - 120**	current study
**postjunctional spikelet peak delay (ms)**	ferret&rat/PFC&VC	14d-2m	**PC=PC**	12	0.80 ± 0.12	0.19 - 1.8	current study
	ferret&rat/PFC&VC	18d, 50d	FS=FS	6	1.20 ± 0.32 **	0.58 - 2.60	current study
	rats/SS&VC	14-18d	FS=FS	10	0.34 ± 0.02		Galarreta, 1999 [1]
	mice/neocortex	2-7m	FS=FS	10	0.26 ± 0.02		Galarreta, 2002 [2]
	rat/SS	20-35d	NGF=NGF	8	0.34 ± 0.09		Simon, 2005 [4]
	rat/SS	20-35d	NGF=FS	6	0.28 ± 0.12		Simon, 2005 [4]
	rat/SS	20-35d	NGF=RS	6	0.32 ± 0.12		Simon, 2005 [4]
**half-duration of postjunctional spikelet (ms)**	ferret&rat/PFC&VC	14d-2m	**PC=PC**	9	16 ± 3	4 - 31	current study
	ferret&rat/PFC&VC	18d, 50d	FS=FS	6	6.8 ± 2.4	0.2 - 12.9	current study
	rat/SS	20-35d	NGF⇒FS	6	9.11 ± 2.95		Simon, 2005 [4]
	rat/SS	20-35d	NGF⇐FS	6	3.80 ± 1.23		Simon, 2005 [4]
**half-duration of postjunctional AP (ms)**	ferret&rat/PFC&VC	14d-2m	**PC=PC**	11	3.0 ± 0.3	2.0 - 4.3	current study

"*" The number of PC pairs with touching or overlapping somata or with negligible separations between somata.

"**" The postjunctional spikelet was induced by a prejunctional AP doublet in two pairs of FS gap junctions, resulting in longer peak delay of the postjunctional spikelet.

1. PFC, prefrontal cortex; VC, visual cortex; SS, somatosensory cortex.

2. PC, pyramidal cell; FS, fast spiking cell; RS, regular spiking cell; LTS, low threshold spiking cell; NGF, neurogliaform cell.

3. References: *(1)* M. Galarreta, S. Hestrin, **Nature** 402, 72 (1999). *(2)* M. Galarreta, S. Hestrin, **PNAS** 99, 12438 (2002). *(3)* M.R. Deans, J.R. Gibson, C. Sellitto, B.W. Connors, D.L. Paul, **Neuron** 31, 477 (2001). *(4)* A. Simon, S. Olah, G. Molnar, J. Szabadics, G. Tamas, **J Neurosci** 25, 6278 (2005). *(5)* J. R. Gibson, M. Beierlein, B. W. Connors, **Nature** 402, 75 (1999).
